# 'Salvage Treatment' of Aggressive Giant Cell Tumor of Bones with Denosumab

**DOI:** 10.7759/cureus.291

**Published:** 2015-07-30

**Authors:** Raju Vaishya, Amit Kumar Agarwal, Vipul Vijay

**Affiliations:** 1 Orthopaedics, indraprastha Apollo Hospital; 2 Orthopaedics, Indraprastha Apollo Hospital

**Keywords:** denosumab, giant cell tumor of bone, rankl

## Abstract

Giant cell tumor of the bone (GCTB) presents as a lytic lesion of epiphyseometaphyseal regions of the long bones usually during the second to the fourth decade with female predilection. Histologically, they are formed of neoplastic mononuclear cells with a higher receptor activator of nuclear factor kappa-B ligand (RANKL) expression responsible for the aggressive osteolytic nature of the tumour. RANKL helps in the formation and functioning of osteoclasts. A newer molecule, Denosumab, is a monoclonal antibody directed against RANKL and thus prevents the formation and function of osteoclasts. Management of refractory, multicentric, recurrent, or metastatic GCTB remains challenging as achieving a tumor-free margin surgically is not always possible. Denosumab may play a crucial role, especially in the management of such difficult lesions. We present three cases of locally aggressive GCTB (involving proximal humerus, sacrum, and proximal femur) that were treated and responded very well to Denosumab therapy.

## Introduction

Giant cell tumor of the bone (GCTB) is a commonly seen bone tumor in clinical practice and represents 4-10% of primary bone malignancies. They usually present as benign or locally aggressive lytic lesions with a predilection for the epiphyseometaphyseal regions of the long bones [1-2]. Histologically, a GCTB is formed of neoplastic ovoid mononuclear cells with a higher receptor activator of nuclear factor kappa-B ligand (RANKL) expression responsible for the aggressive osteolytic nature of the tumor [3-4]. RANKL helps in the formation and functioning of osteoclasts. Stimulation of osteoblasts in bone metastasis increases the expression of RANKL by tumour-secreted factors, which binds osteoprotegerin, resulting in increased bone resorption. A newer molecule, denosumab, is a novel monoclonal antibody directed against RANKL and thus prevents the formation and function of osteoclasts.

Surgical resection or extended curettage and bone cementing are the usual treatment options for GCTB, but for unresectable cases, treatment options so far have been limited. The main issue with the surgical resection is the high recurrence rate: 27%–65% after isolated curettage; 12%–27% after curettage with adjuvant, such as high-speed burr, phenol, liquid nitrogen, or polymethyl methacrylate (PMMA); and 0%–12% after en bloc resection [5-6]. A few studies have also shown intralesional injections as a possible means of treatment for selected patients, which may be an intralesional steroid, intralesional calcitonin, intralesional interferon, or intralesional bisphosphonates [7-9]. Similarly, management of refractory, multicentric, and recurrent or metastatic GCTB remains challenging [10]. Achieving a disease-free and clean tumor margin in these cases may be a surgical challenge. Their aggressive nature makes it difficult to achieve surgical clearance, especially in the areas that are not easily amenable to surgery. Denosumab may play a crucial role, especially in the management of such difficult lesions. Being a relatively new molecule, denosumab and its role in the management of GCTB is not universally known to orthopedic surgeons or oncologists at present [11].

We present three cases of locally aggressive GCTB (involving the proximal humerus, sacrum, and proximal femur) treated with denosumab therapy.

## Case presentation

The Indraprastha Apollo Hospital Ethics Committee reviewed and approved this study. Informed patient consent was obtained from all patients involved in this study.

Patient ages ranged from 19 to 32 years. All three patients were male. The reason for denosumab therapy in these patients included unsalvageable lesions or refusal of operative treatment for the recurrence. These cases were given injectable denosumab in the dose of 120 mg subcutaneously as a loading dose, followed by the same dose on 8th and 15th days and then every month for six months. The response to denosumab therapy was noted both clinically and radiologically every month.

### Case 1

**Figure 1:**
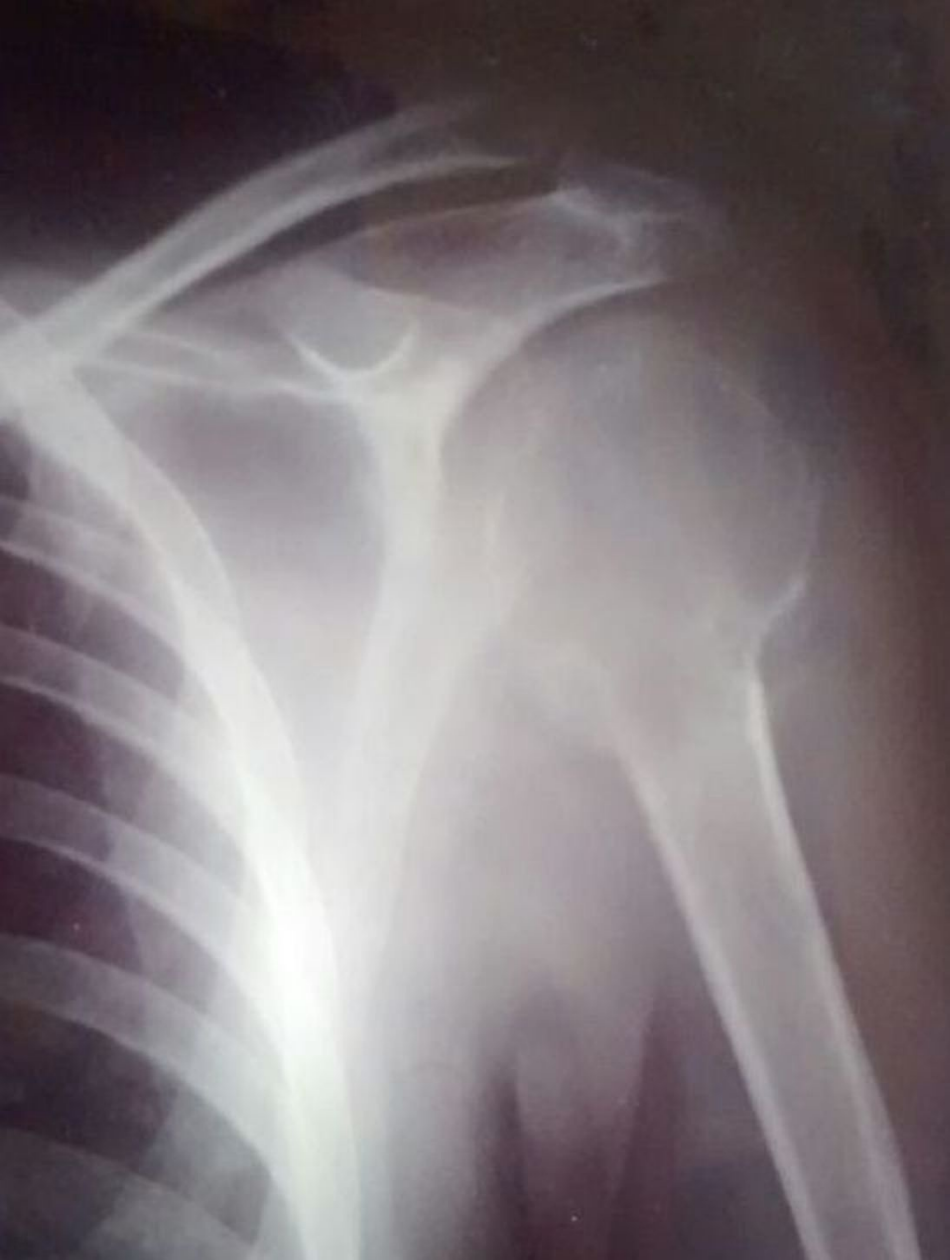
Anteroposterior radiograph of the left shoulder (Case #1) showing a large lytic lesion involving the whole humeral head and proximal humerus with indistinct cortices and uniform radiolucency.

A 27-year-old young man presented with complaints of left shoulder pain of one year's duration. Initial imaging revealed a lytic lesion involving the whole of the left humeral head. There was an impending pathological fracture at the surgical neck of the humerus (Figure 1).

**Figure 2:**
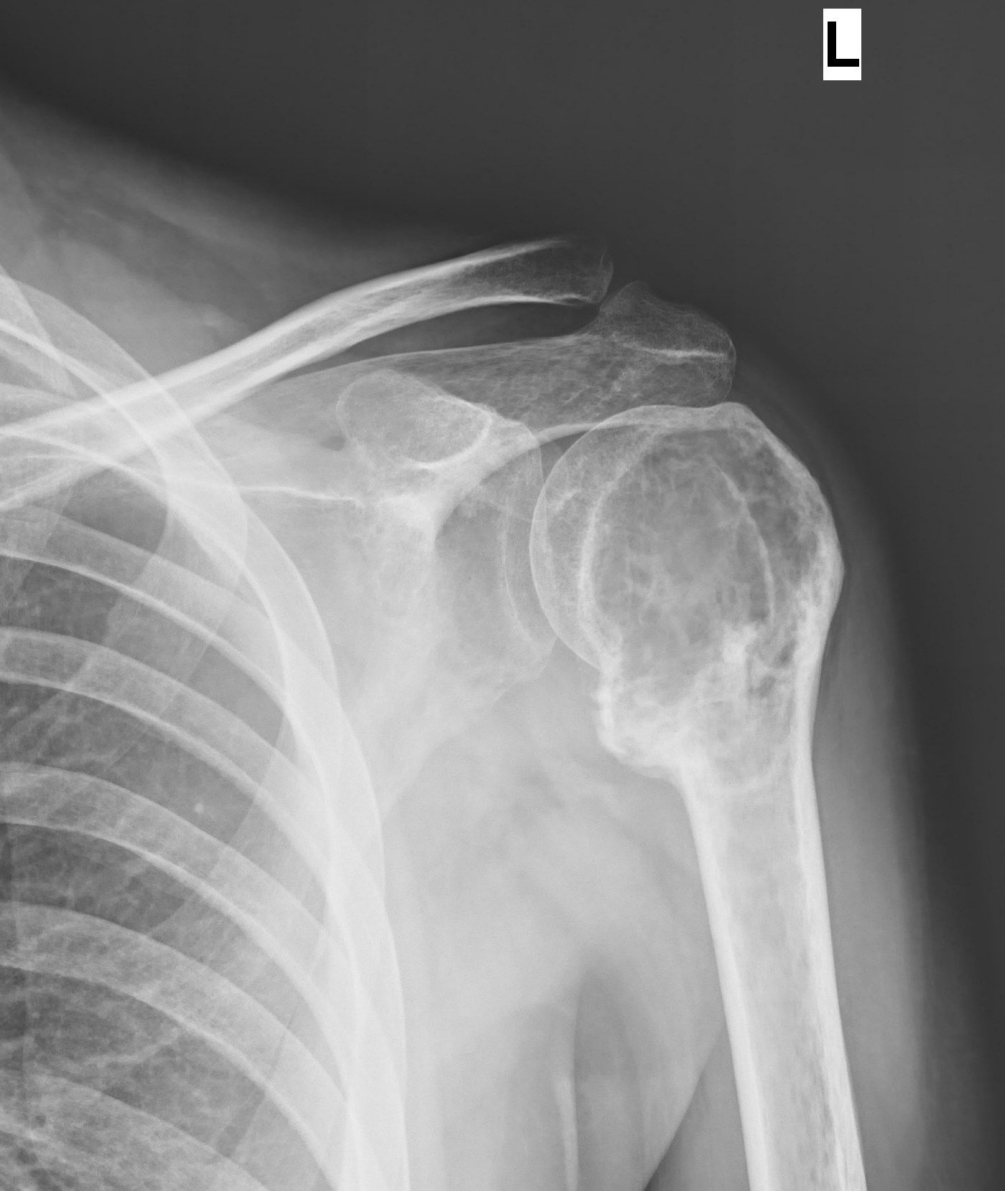
Post-denosumab therapy radiograph of the left shoulder (Case #1) showing patchy ossification and sclerotic rimming of the lesion in the proximal humerus.

A biopsy confirmed a diagnosis of GCTB (Figure 2).

**Figure 3:**
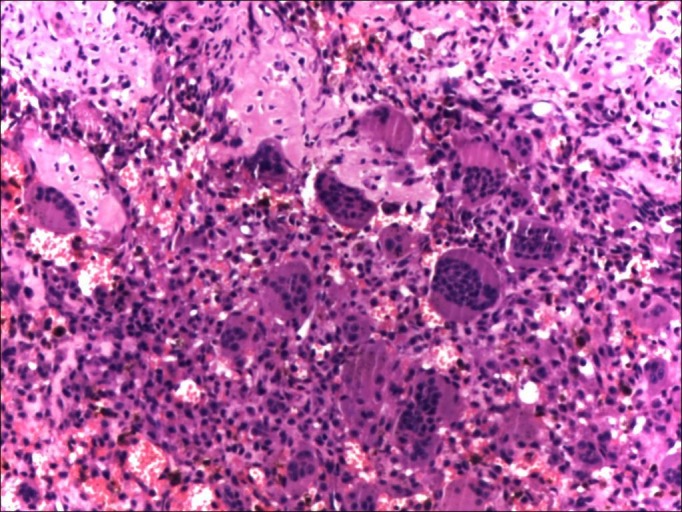
Pre-denosumab histopathological picture from the left proximal humerus (Case #1) showing typical features of any giant cell tumor, including numerous multi-nucleated giant cells.

Extended curettage and bone cementing could not be done as sufficient bone stock was not available for the procedure and the lesion appeared to be unsalvageable. Hence, he was put on denosumab therapy. There was a dramatic response clinically as well as radiologically. At the end of six months, extended curettage and cementing were done as the lesion became more contained and salvageable (Figure 3).

**Figure 4:**
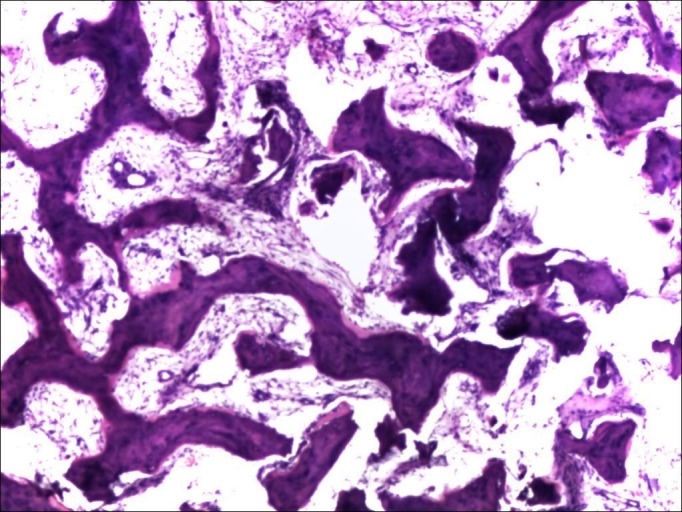
Post-denosumab histopathological picture (Case #1) showing osseo-fibrous conversion of the lesion with disappearance of multinucleated giant cells.

Histopathological examination showed fragments of dense fibro-osseous tissue, comprised of abundant osteoid separated by mildly cellular fibrous tissue. Focal calcification and islands of woven bone were also present at places. These features were consistent with effects of denosumab therapy in a known case of GCTB (Figure 4).

### Case 2

**Figure 5:**
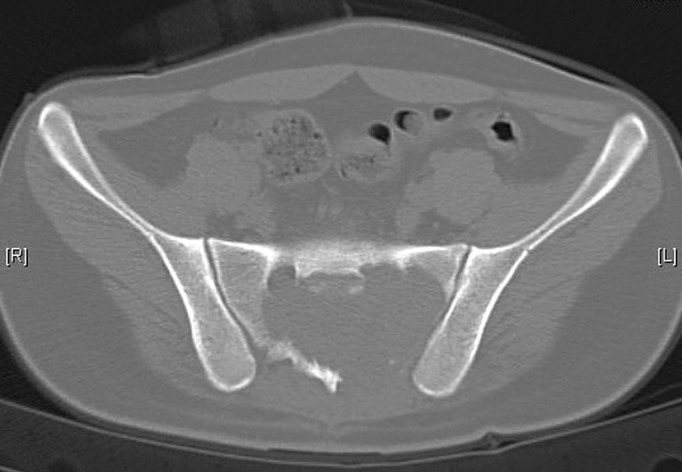
Computer tomography (CT scan) showing a large lytic lesion involving the sacrum (Case #2).

A 19-year-old man presented with pain in the lower back radiating to both lower limbs and difficulty in passing stool and urine over the preceding four months. He was diagnosed as having an expansile lesion in the sacrum, which was diagnosed as GCT on biopsy. He underwent spinal decompression and resection of the tumor in the S2-3 region. However, he continued to be symptomatic following the surgery. On repeated MRI after one month, there was evidence of a persistent lesion (Figure 5).

**Figure 6:**
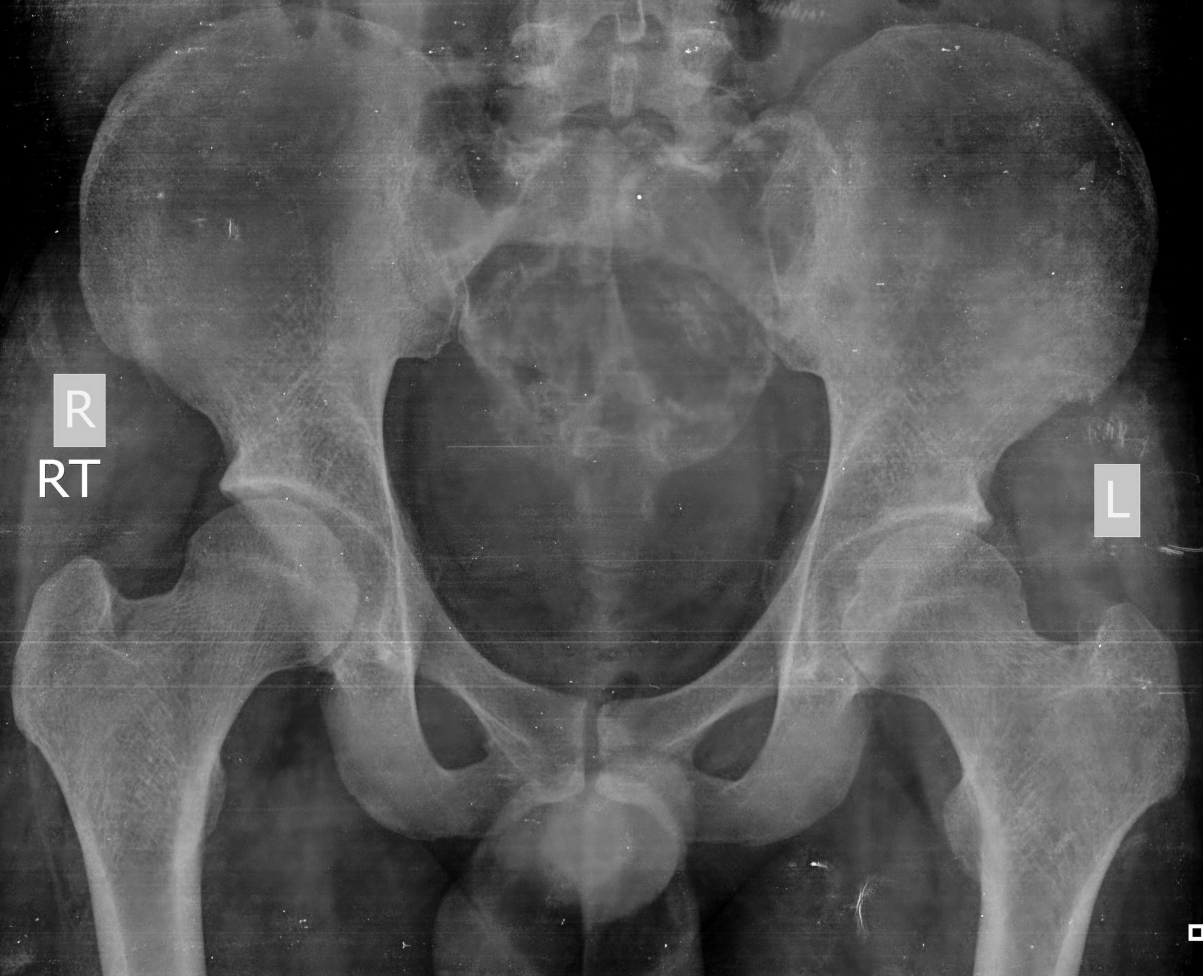
Anteroposterior radiograph of the pelvis showing patchy calcification and ossification with peripheral sclerosis of the tumor, indicating improvement after denosumab therapy (Case #2).

He was therefore given denosumab therapy. The pain and swelling had started to regress after one month of therapy and his symptoms continued to improve at the completion of therapy at six months. On repeated x-ray, the tumor size had decreased and there was a dramatic improvement in the pain (Figure 6). Radiological healing of the lesion was visible six weeks after therapy and was significantly apparent at the completion of therapy.

### Case 3

**Figure 7:**
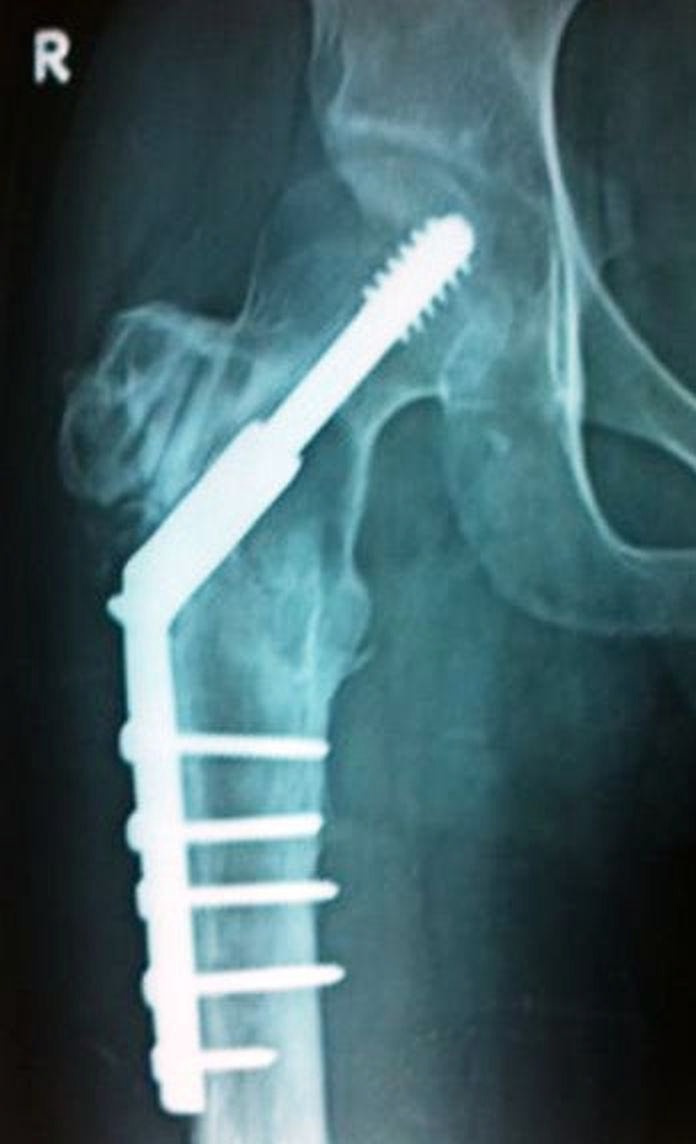
Anteroposterior radiograph of the hip (Case #3) after curettage, bone grafting and plate fixation for a histologically confirmed GCT of the proximal left femur.

A 32-year-old male underwent curettage, bone grafting, and plate fixation (Figure 7) for a histologically proven GCT of the proximal left femur two years previously.

**Figure 8:**
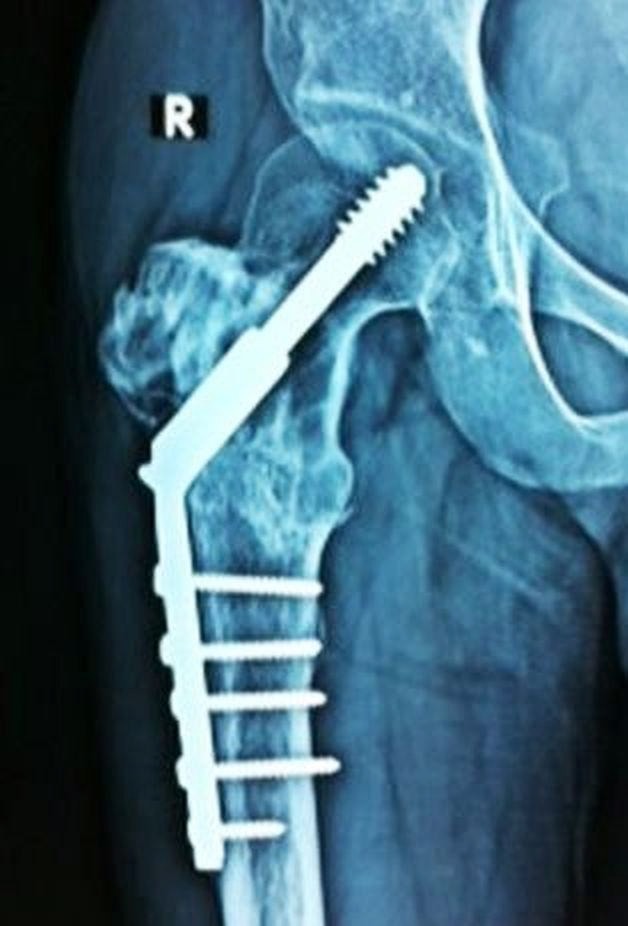
Anteroposterior radiograph of the left proximal femur (Case #3) showing healing of the lesion after 6 months of denosumab therapy.

He then presented with pain around the operative site. The plain radiograph revealed evidence of a recurrence of GCT at the previously operated site. The patient was unwilling to undergo further surgery so we started him on denosumab therapy. After six months of therapy, the patient was much better clinically as well as radiologically (Figure 8).

The radiological evidence of healing of the lesions included a) cortical thickening (sclerotic rimming) and b) increased calcification and ossification inside the tumor, due to the reactive bone formation.

## Discussion

A GCTB is benign but locally aggressive bone tumor. Microscopically, the hallmark feature of a GCT is the presence of multiple multinucleated giant cells (formed from a fusion of mononuclear cells). A GCTB may present with local recurrence, multicentric or complex lesions that cannot be removed surgically without causing significant morbidity. A GCTB usually occurs during the second to fourth decades of life [12], with a female preponderance (1:1.2 to 1:1.5); however, in our three cases, all were males (Table 1).

**Table 1:** Brief clinical history of patients with GCTB, site of lesion and response to the denosumab therapy

Serial No.	Age/Sex	Site of Lesion	Duration of Symptoms/ Recurrence	Reason for Denosumab Therapy	Response to Denosumab Therapy
1	27 yrs/M	Proximal humerus	1-year	Non -salvageable lesion	Good, extended curettage and cementing done
2	19 yrs/M	Sacrum	4 months	Recurrence, no response to multiple selective arterial embolization and regular bisphosphonates	Good
3	32 yrs/M	Greater trochanter left	2 years	Recurrence, patient not willing for surgery	Good

Various forms of treatment have been used for the treatment of GCTB. The current preferred treatment for Stage I and II is extended curettage of the tumor, for Stage III an en bloc excision or amputation with reconstructive procedures, if required, and for surgically inaccessible lesions, radiotherapy is advised. The results of all these procedures are not optimal as they are associated with recurrence and other complications. Wide resection is associated with a lower recurrence rate compared with intralesional surgery (5% vs. 25%), but it involves the complex problem of reconstruction and associated morbidity [13]. Radiotherapy can be used for local control of unresectable tumors. However, radiotherapy is associated with sarcomatous changes and other complications [14]. Chemotherapy and interferon-α have shown a good response in the treatment of GCTB in a few studies, but their efficacy is still not proven. Antitumor activity of interferon-α has been shown by few case reports, but this efficacy is at the cost of increased toxicity [15].

Denosumab has been recently used for the palliative treatment of GCTB [13-14]. However, due to lower levels of evidence available so far pertaining to this topic (i.e., case reports, retrospective reviews, etc.), the validity of these data are limited and hence require further research and documentation related to its efficacy and adverse events. In a meta-analysis, denosumab was found to be more effective than bisphosphonates and placebo in delaying skeletal complications during treatment of bone metastases secondary to solid tumors [16]. Denosumab is a monoclonal antibody that inhibits the action of RANKL, thereby reducing the differentiation, activation, and survival of osteoclasts. Denosumab inhibits osteoclast-mediated osteolysis and suppresses bone turnover in GCTB [17]. Inhibiting RANKL in turn inhibits osteoclast-like giant cells and their associated mononuclear cells in GCTB. Denosumab is primarily indicated for the prevention of skeletal-related events in patients with bone metastases from solid tumors [18]. Complications of denosumab reported in the literature include hypersensitivity reactions, urinary tract infection, respiratory tract infection, dyspnea, sciatica, cataracts, constipation, hyperhidrosis, myalgia, hypocalcaemia, hypophosphatemia, atypical femur fracture, and osteonecrosis of the jaw (ONJ). Complication of ONJ is known, although uncommon in cancer patients after denosumab therapy. Osteonecrosis of the jaw has been reported in patients receiving denosumab at a dose as low as 60 mg every six months for osteoporosis. Daily calcium and vitamin D supplements are recommended to prevent these adverse events [19-20]. There were no serious short- or long-term complications noted in any of our patients.

These cases further enhance the evidence of efficacy and safety of denosumab in challenging cases of GCTB which are not immediately amenable to surgery. Moreover, we agree that, on histopathological analysis, the tissue specimens from patients with GCTB on denosumab therapy confirmed a significant reduction of giant cells and reduction of cellular and proliferative tumor stroma. A radiological comparison clearly shows the improvement in the lytic picture with peripheral healing by increased sclerosis of the cortices of an involved bone and reactive bone formation in the tumor [21]. Recent advances and research have led to better understanding of the biological mechanisms of a pathological condition leading to the development of targeted therapy at a molecular level. Denosumab is a newer treatment option for unresectable and recurrent GCTB.

However, we believe that before advocating the regular use of denosumab for GCTB a few questions need to be answered regarding an optimal schedule of therapy, its dosing and its effect on the immature skeleton, pregnancy, and in lactating women, etc. A further question arises as to whether denosumab can be used for definitive therapy of GCTB and if it can reduce the extent of surgery or recurrence rates after definitive surgery. In our first case, denosumab acted as a palliative agent and helped in surgical curettage by reducing the size of the tumor, whereas, in second case, it was the only viable option as the lesion was not amenable for surgery. Long-term follow-up of patients treated with denosumab is not available in the literature. Early detection of recurrence is difficult as there is no specific tumor marker for giant cell tumors. The dilemma of whether the effect of denosumab on GCTB is temporary or definitive remains unanswered. However, there is one case report published recently showing rapid recurrence after cessation of long-term denosumab therapy [22]. More long-term multicentric studies with large sample population are required in the future to answer these questions.

## Conclusions

In our experience, denosumab is a newer treatment option for unresectable and recurrent GCTB with good clinical as well as radiological outcomes. It aids well in the reduction of the surgical trauma by reducing the amount of surgical resection and may be helpful with medically compromised patients. However, long-term multicentric studies with large sample populations are required in the future to clearly define its role for the management of primary GCTB as well as to find the answers regarding the optimal scheduling, patient selection, use in the adjuvant setting, and long-term toxicity concerns.
